# Genome Features and Biochemical Characteristics of a Robust, Fast Growing and Naturally Transformable Cyanobacterium *Synechococcus elongatus* PCC 11801 Isolated from India

**DOI:** 10.1038/s41598-018-34872-z

**Published:** 2018-11-09

**Authors:** Damini Jaiswal, Annesha Sengupta, Sujata Sohoni, Shinjinee Sengupta, Ambarish G. Phadnavis, Himadri B. Pakrasi, Pramod P. Wangikar

**Affiliations:** 1 Department of Chemical Engineering, Indian Institute of Technology Bombay, Powai, Mumbai, 400076 India; 2 DBT-PAN IIT Centre for Bioenergy, Indian Institute of Technology Bombay, Powai, Mumbai, 400076 India; 3 Department of Biology, Washington University, St. Louis, MO 63130 USA; 4 Department of Energy, Environmental and Chemical Engineering, Washington University, St. Louis, MO 63130 USA; 5 Wadhwani Research Centre for Bioengineering, Indian Institute of Technology Bombay, Powai, Mumbai, 400076 India

## Abstract

Cyanobacteria provide an interesting platform for biotechnological applications due to their efficient photoautotrophic growth, amenability to genetic engineering and the ability to grow on non-arable land. An ideal industrial strain of cyanobacteria would need to be fast growing and tolerant to high levels of temperature, light, carbon dioxide, salt and be naturally transformable. In this study, we report *Synechococcus elongatus* PCC 11801, a strain isolated from India that fulfills these requirements. The physiological and biochemical characteristics of PCC 11801 under carbon and light-limiting conditions were investigated. PCC 11801 shows a doubling time of 2.3 h, that is the fastest growth for any cyanobacteria reported so far under ambient CO_2_ conditions. Genome sequence of PCC 11801 shows genome identity of ~83% with its closest neighbors *Synechococcus elongatus* PCC 7942 and *Synechococcus elongatus* UTEX 2973. The unique attributes of PCC 11801 genome are discussed in light of the physiological characteristics that are needed in an industrial strain. The genome of PCC 11801 shows several genes that do not have homologs in neighbor strains PCC 7942 and UTEX 2973, some of which may be responsible for adaptation to various abiotic stresses. The remarkably fast growth rate of PCC 11801 coupled with its robustness and ease of genetic transformation makes it an ideal candidate for the photosynthetic production of fuels and chemicals.

## Introduction

Cyanobacteria are the only group of prokaryotes that perform oxygenic photosynthesis. They play an important role in aquatic ecosystems as primary producers because of their ability to fix CO_2_ into reduced carbon substrates using solar energy^1^. The cyanobacterial phylum comprises diverse groups of organisms capable of inhabiting extreme environmental niches with a high degree of success. Moreover, cyanobacteria can grow on non-arable land and wastewater thereby minimizing competition with food and feed. The short life cycle, minimal nutrient requirements, high photosynthetic efficiency and amenability to genetic modifications make this photoautotrophic group of prokaryotes an attractive platform for biotechnological applications^2,3^. In a future biorefinery, engineered cyanobacteria may be deployed for the production of biofuels and platform chemicals using CO_2_ as feedstock and by harnessing solar energy. While such processes may not be commercially viable at present, significant promise lies in the proof of concept studies that demonstrate pathway engineering of cyanobacteria to produce a number of chemicals^4^. Indeed, laboratory strains of cyanobacteria such as *Synechococcus elongatus* PCC 7942 and *Synechocystis* sp. PCC 6803 (henceforth referred to as PCC 7942 and PCC 6803, respectively) have been engineered to produce some compounds such as alkanes^5^, hydrogen^6^, ethanol^7^, butanol^8^, 2,3-butanediol^9^, acetone^10^, ethylene^11^, fatty acids^12^, and isoprene^13^. Metabolic flux analysis of the model cyanobacterial strains has provided useful information on strain engineering^14,15^.

There are several technical challenges that limit the use of cyanobacteria in industrial applications despite their great biotechnological potential. First, the laboratory strains of cyanobacteria such as PCC 7942 and PCC 6803 grow at rates that are an order of magnitude slower than the heterotrophic workhorse *Escherichia coli*. Second, the presence of multiple genome copies in most cyanobacteria makes the process of genetic engineering and chromosomal segregation a time consuming one^16^. Synthetic biology toolkit development in cyanobacteria has lagged behind the heterotrophic counterparts^17^. Detailed characterization of native parts of the model strains PCC 6803 and *Synechococcus* sp. PCC 7002 (henceforth PCC 7002) has been reported only recently^18,19^.

Moreover, the model strains of cyanobacteria may not be tolerant to the outdoor cultivation conditions or even to the products, the biosynthesis of which can be engineered in these bacteria. A recently reported non-model strain, *S*. *elongatus* UTEX 2973 (henceforth UTEX 2973) is a potential host for biotechnological applications due to its fast growth, but it is not naturally transformable. Another fast growing and naturally transformable model cyanobacterium PCC 7002 cannot grow without external supplementation of vitamin B_12_^20^ that would subsequently increase the production cost significantly.

A fast-growing and genetically amenable cyanobacterium that can tolerate wide ranges of light intensities, temperature, CO_2_ levels, and salinity would be an ideal candidate for metabolic engineering applications. In this study, we describe the isolation, identification, physiological and genomic characterization of a novel cyanobacterium, *Synechococcus elongatus* PCC 11801 (henceforth PCC 11801). The growth rate of PCC 11801 is significantly greater than other reported model cyanobacterial strains such as PCC 7942, PCC 7002, and PCC 6803 and comparable to UTEX 2973^16^. Moreover, the isolated strain is naturally transformable and tolerant to high light, temperature, CO_2_ and sea salt concentration. Genome characterization reveals that PCC 11801 has a number of genes that impart unique characteristics to strive under extremes of environmental conditions. Our study indicates that PCC 11801 can serve as a chassis for photosynthesis coupled biosynthesis.

## Results and Discussions

### Isolation, Identification and Phylogenetic Assignment

The axenic culture of PCC 11801 was isolated from water samples collected from Powai Lake, located in an urban area of Mumbai, India (19.1273°N, 72.9048°E) using a combination of isolation techniques described in methods section. The water samples were collected on 2^nd^ June 2015 to coincide with the end of summer but before the beginning monsoon. The temperature at the surface of the water body was 34 °C and pH was 7.5 at the time of sample collection. For selection of potential freshwater, saltwater, and mesohaline cyanobacteria, the samples were inoculated in three media namely, BG-11, ASN-III and BG-11 + ASN-III (a 50:50 mixture, v/v). Visible blue-green growth was observed within two days of inoculation in the BG-11 medium. A marginal growth was seen in BG-11 + ASN-III medium and no growth was observed in ASN-III medium in 2 days. Hence, the BG-11 medium was used for subsequent isolation procedure.

**Figure 1:**
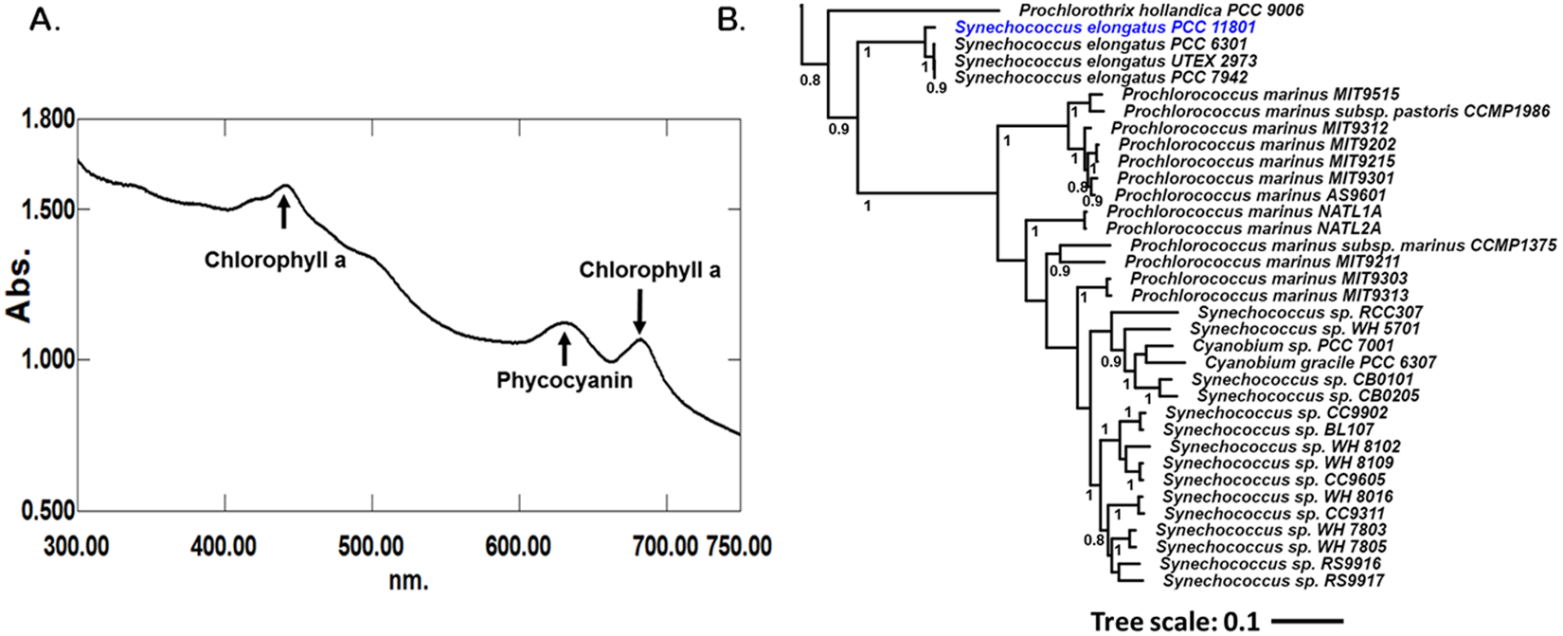
Identification and Phylogenetic Assignment. (**A**) An absorbance spectrum (300–750 nm) recorded for PCC 11801 showing the characteristic peak of phycocyanin at 620 nm and (**B**) A part of the phylogenetic tree constructed based on concatenated protein sequences of 29 house-keeping genes, showing PCC 7942 and 6301 as the closest neighbors of PCC 11801. Nodes supported by bootstrap values of >0.7 are indicated.

The isolation conditions were chosen to select cyanobacterial strains that can tolerate a temperature of >38 °C and light intensity of >400 µmole photons.m^−2^.s^−1^. Treatment of cultures with kanamycin and cycloheximide in dark allowed growth inhibition of heterotrophic prokaryotic and eukaryotic organisms respectively^21,22^. Serial dilution and streaking on BG-11 agar plates facilitated the physical separation of other contaminating bacteria. Axenic cultures of 8 morphologically distinct cyanobacterial strains were isolated, all of which grew faster than the model strains of PCC 7942 and PCC 6803. Preliminary growth characterization led to the selection of PCC 11801 that had the highest growth rate. The presence of cyanobacteria was confirmed by a distinct peak at 620 nm corresponding to phycocyanin in the whole cell absorption spectrum of the culture (Fig. 1A) recorded in UV-visible range. The genus of the isolated strain was identified using 16S rRNA sequencing and subsequent comparison with database sequences using BLAST. The top 10 hits belonged to the genus *Synechococcus* with a sequence similarity of >95% and hence the genus *Synechococcus* was tentatively assigned to the isolate. Subsequently, the whole genome was sequenced, which enabled a more precise phylogenetic placing of the strain. This was achieved by constructing a phylogenetic tree by using concatenated sequences of 29 conserved proteins involved in replication, transcription and translation that are less likely to be laterally transferred^23–25^. The respective protein sequences were obtained from the sequenced and annotated whole genome of PCC 11801. The results obtained with this analysis (Figs 1B and S2) are broadly in agreement with 16S rRNA phylogeny (Fig. S1). However, bootstrap confidence at nodes was far better with the 29 conserved proteins than with the 16S rRNA sequence alone (Figs S1 and S2). PCC 11801 forms a monophyletic clade with *Synechococcus elongatus* PCC 7942 and 6301 in both analyses suggesting that the isolated organism belongs is *Synechococcus elongatus*. The isolate has been deposited to Pasteur Culture Collection of Cyanobacteria (PCC) as *Synechococcus elongatus* PCC 11801.

### Fast growth and tolerance to abiotic factors

**Figure 2:**
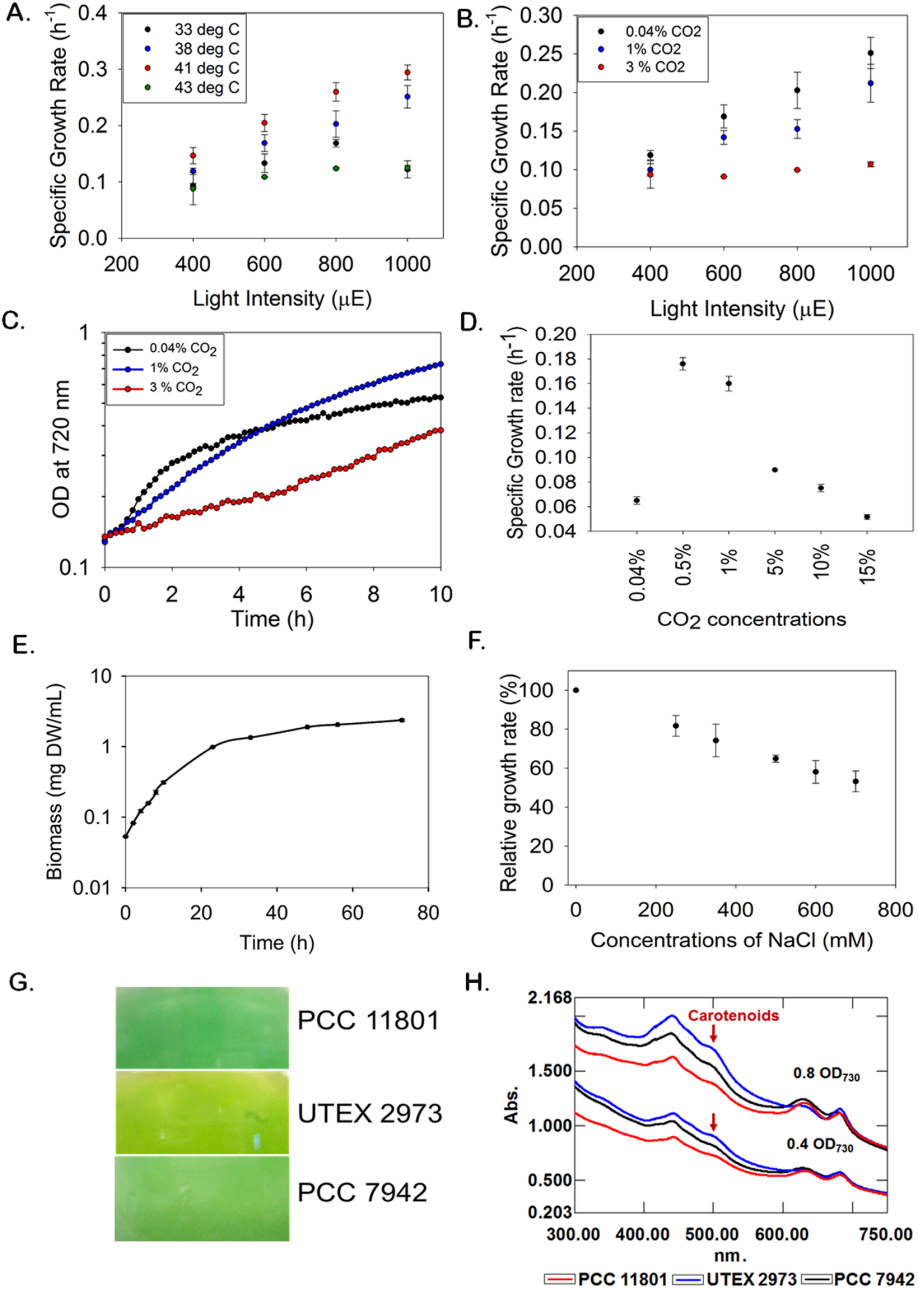
Physiological characterization of PCC 11801. Comparison of specific growth rates of PCC 11801 at (**A**) varying temperatures and light intensities when ambient air is bubbled in a multi-cultivator (MC) and (**B**) varying CO_2_ and light conditions in MC at 38 °C, (**C**) Comparison of growth profiles under varying CO_2_ conditions at 1000 µmole photons.m^−2^.s^−1^ and 38 °C in MC, (**D**) Comparison of specific growth rates in SF at varying CO_2_ concentrations in an incubation chamber (400 µmole photons.m^−2^.s^−1^, 38 °C), (**E**) The biomass of PCC 11801 versus time at 400 µmole photons.m^−2^.s^−1^, 38 °C and 0.5% CO_2_ (**F**) Tolerance of PCC 11801 to varying NaCl concentrations (**G**) Comparison of the phenotype of cultures of PCC 11801, UTEX 2973 and PCC 7942 grown under similar conditions in SF and (**H**) Comparison of whole cell spectrum of PCC 11801, UTEX 2973 and PCC 7942.

PCC 11801 showed a doubling time of 2.3 hours under its optimal growth conditions that included cultivation at 41 °C, light intensity of 1000 µmole photons.m^−2^.s^−1^ and bubbling of ambient air (Fig. 2A). The growth rate of PCC 11801 is greater than those of the widely used model strains such as PCC 7002, PCC 7942, PCC 6803^16^ and comparable to the fastest growing cyanobacteria reported till date, UTEX 2973, which shows a doubling time of 2.1 h at 3% CO_2_ and light intensity of 500 µmole photons.m^−2^.s^−1^ ^16^. Unlike other cyanobacteria that achieve their optimal growth at higher CO_2_ levels^16^, PCC 11801 shows optimal growth when ambient air is bubbled in the tubes of a multi cultivator (MC) (Fig. 2B). This growth rate is the highest reported till date for any cyanobacterium under ambient CO_2_ and without any additional supplementation in the growth medium such as vitamins, sodium bicarbonate, etc. The growth rate of PCC 11801 was highest under ambient CO_2_ at all light intensities and temperature conditions (Fig. 2A,B and Table S1). Growth profiles under 0.04% and 1% CO_2_ conditions (Fig. 2C) show that although the growth rate during exponential phase is greater under 0.04% CO_2_, the exponential growth phase lasts for a longer period under 1% CO_2_. The culture continues to grow with the constant growth rate for longer duration and accumulates higher biomass at 1% CO_2._ Thus, the carbon limitation resulted due to higher cell density is alleviated at elevated CO_2_ conditions.

PCC 11801 could tolerate up to 15% CO_2_ level in the incubation chamber when grown in a shake flask (SF) (Fig. 2D). Surprisingly, we noticed that the growth of PCC 11801 was optimal at 0.5–1% CO_2_ in SF (Fig. 2D), unlike in MC (Fig. 2A,B). This may be because of better availability of CO_2_ and nutrients due to direct bubbling of air in MC allowing proper cell mixing, aeration, and nutrient uptake. Slower growth in SF is overcome at higher CO_2_ concentrations as depicted by rapid growth at 0.5–1% CO_2_ (Fig. 2D). The time profile of dry biomass of PCC 11801 at 0.5% CO_2_ in SF is shown in Fig. 2E. The phenotypic differences observed in growth profiles of PCC 11801 between MC and SF suggest that the rapid growth is also determined by the availability of CO_2_ to cells rather than only the CO_2_ concentration in the air. PCC 11801 is also capable of tolerating sea salt concentration although the growth rate decreases with increasing salt concentrations (Fig. 2F). On the other hand, the model strain PCC 7942 cannot grow at these sea salt concentrations^26^.

We also observed the phenotypic differences between the cultures of PCC 11801, PCC 7942 and UTEX 2973 growing under similar conditions (0.04% CO_2_, 38 °C and 400 µmole photons.m^−2^.s^−1^ in SF). UTEX 2973 and PCC 7942 exhibited yellow-green coloration while PCC 11801 remained dark blue-green at similar cell densities (Fig. 2G). The whole cell spectra recorded at similar optical densities showed higher carotenoid content in UTEX 2973 and PCC 7942 as compared to PCC 11801. This was depicted by the absorbance at 520 nm corresponding to absorption maxima of carotenoids (Fig. 2H). It has been reported that the upregulation of carotenoids in high light is typically a result of photo-protective mechanism and helps prevent cellular damage^27,28^. A relatively higher requirement of carotenoids for photoprotection by UTEX 2973 and PCC 7942 compared to PCC 11801 demonstrates the intrinsic capability of PCC 11801 to tolerate high light. This might be because of ecological conditions of the habitat from where PCC 11801 was isolated. Thus, PCC 11801 may be a suitable candidate for field applications where cultures are required to grow under bright sunlight.

### Biochemical characterization under different CO_2_ conditions

**Figure 3:**
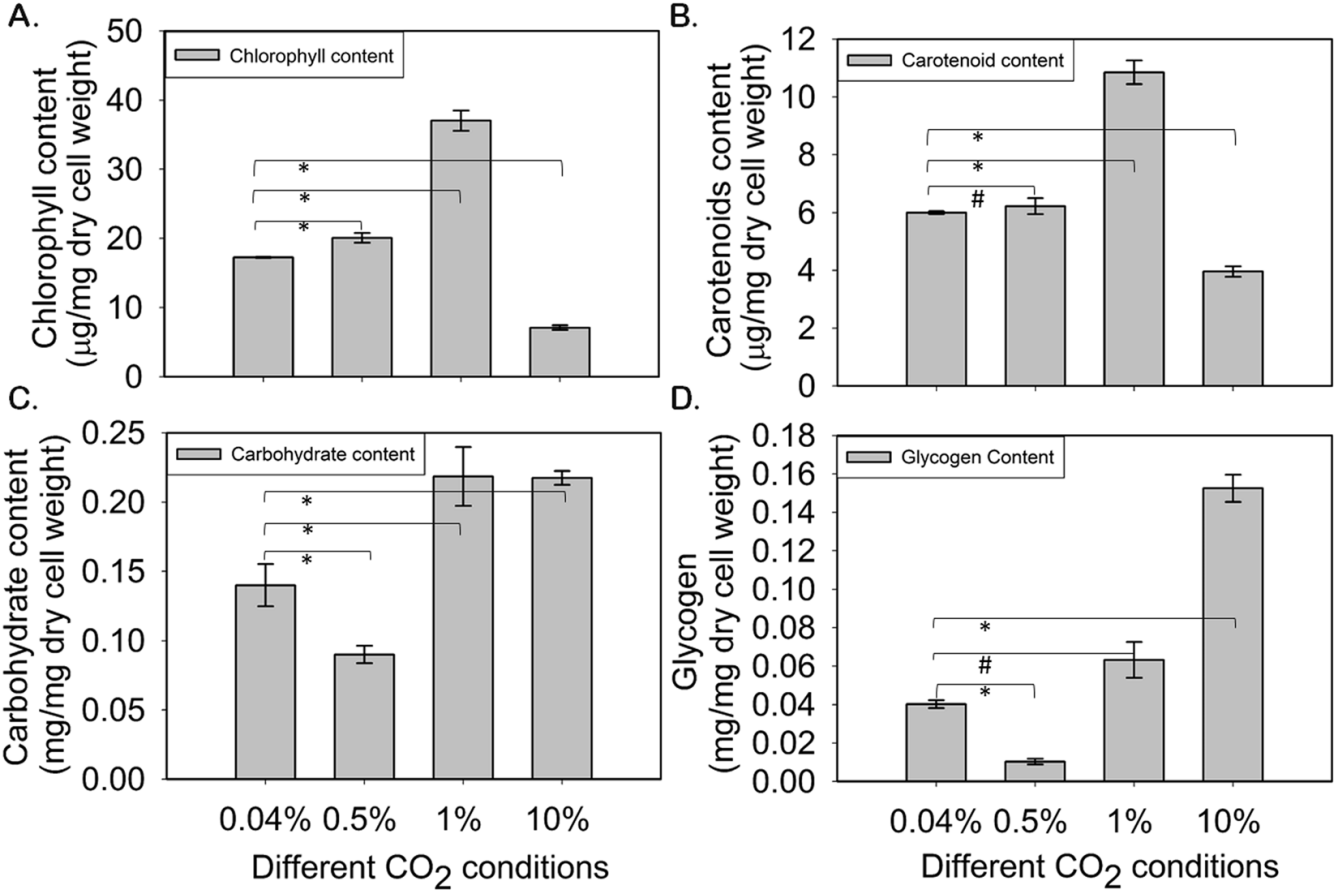
Biochemical composition analysis under varying CO_2_ conditions. (**A**) Chlorophyll (**B**) carotenoid (**C**) carbohydrate and (**D**) glycogen content of PCC 11801 under 0.04%, 0.5%, 1% and 10% CO_2_ in air. ‘*’ denotes that the results are significant with a p-value < 0.05 with t-test while ‘#’ denotes statistically insignificant differences.

The biochemical characterization of PCC 11801 was performed under ambient and elevated CO_2_ conditions (0.5%, 1% and 10%) in SF at 38 °C and 400 µmole photons.m^−2^.s^−1^. It was observed that the chlorophyll and carotenoid contents of the cells were marginally higher at 0.5% CO_2_ as compared to 0.04% CO_2_. However, there was a 2-fold increase in the chlorophyll and carotenoids content at 1% CO_2_ and a significant decrease (2.4-fold in chlorophyll and 1.5-fold in carotenoid, respectively) at 10% CO_2_ (Fig. 3A,B)_._ The increase in pigment content at 1% CO_2_ is indicative of cellular response to trap more light to improve the photosynthetic performance in response to better availability of inorganic carbon. The decrease in the pigment content at 10% CO_2_ may be associated with cellular stress or degradation of excessive pigments that are not required for light harvesting^29^.

The total carbohydrate and glycogen contents were the lowest under the best growth condition (0.5% CO_2_) as shown in Fig. 3C,D. Although there are no reports which directly correlate the growth rate and glycogen content, a recent study shows that glycogen synthesis and degradation may have a role in energy balancing mechanism during varying growth phases in *Synechocystis* sp. PCC 6803^30^. The rapid growth is marked by a high energy charge (ratio of ATP/ATP + ADP) in the cells^30^. Furthermore, a high energy charge also correlates with lower glycogen levels^30^ possibly because glycogen storage is also an energy consuming process. Thus, glycogen synthesis is marked by lower energy charge in the cells that might not be a favorable process during rapid growth. Additionally, glycogen synthesis by ADP-glucose phosphorylase (AGPase) coded by *glgC* is allosterically activated by 3-phosphoglycerate (3PGA), a product of carboxylating reaction by RuBisCO^31^. The downregulation of RuBisCO under high CO_2_ thus would also lead to decreased AGPase activity and glycogen synthesis. We believe that low glycogen phenotype in PCC 11801 during rapid growth (Fig. 3D) is a result of these regulatory mechanisms.

There is a significant increase in glycogen and total carbohydrates at very high CO_2_ condition (10% CO_2_). It can be noted from Fig. 2D that PCC 11801 grows much slower under this condition. Thus high glycogen storage might be an energy balancing mechanism to dissipate excess energy and carbon that could not be used for growth under stress conditions. It is also noticeable that the total carbohydrate content remains constant at 1% and 10% CO_2_ conditions. However, the glycogen comprises 27% and 68% of total carbohydrates at 1% and 10% CO_2_ respectively. This suggests that carbohydrate other than glycogen that might be required for cell growth and maintenance must have increased at 1% CO_2_.

### Structural analysis using TEM and cryo-SEM

**Figure 4:**
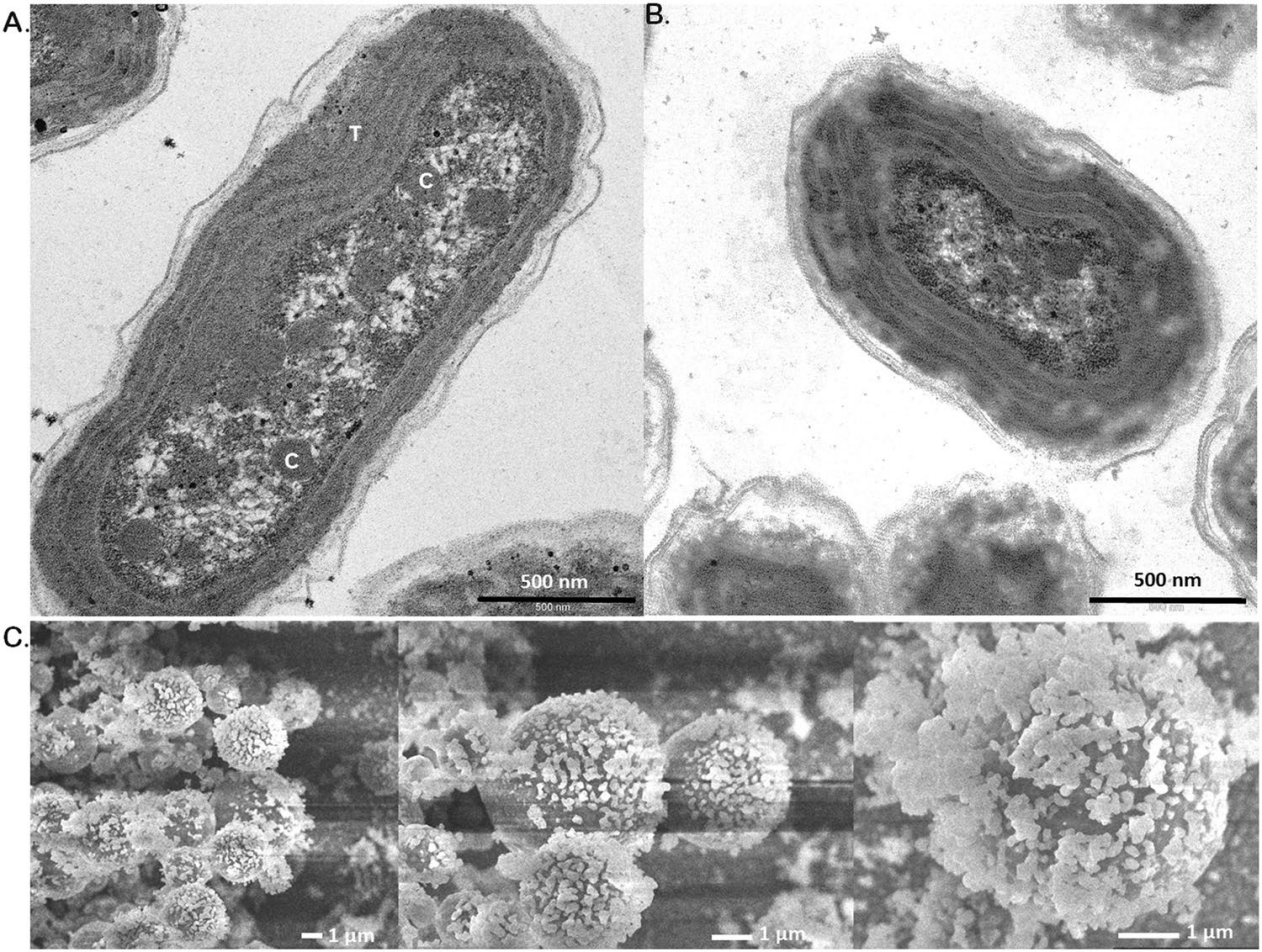
Electron microscopic analysis of *Synechococcus elongatus* PCC 11801. Transmission electron micrographs of PCC 11801 under (**A**) 0.04% CO_2_ and (**B**) 1% CO_2_ in air conditions. (**C**) Scanning electron micrographs of PCC 11801 cells grown under 0.04% CO_2_ showing the presence of exopolysaccharides on the cell surface. Scale as indicated on the respective panel. In panel A, the labels C and T represents carboxysomes and thylakoid membranes respectively.

The subcellular structure of PCC 11801 was studied using transmission electron microscopy. The cells were rod-shaped, with an average length of 2.5 µm and a width of 1.4 µm. The cell wall boundary was found to be irregular. The cell envelope was followed by approx. 4 thylakoid membranes. Polyphosphate bodies and carboxysomes were located in the cytoplasm. To study changes in subcellular organization in response to CO_2_, TEM was performed using the cells grown in 0.04% and 1% CO_2_ in SF. It was found that there was a 1.9-fold reduction in the length of cells grown under 1% CO_2_ (Fig. 4A,B). The fold change was calculated using mean the length of cells under 0.04% and 1% CO_2_ from 69 and 78 individual cell measurements, respectively (Table S2). The results were found to be significant with a p-value < 0.01 with a paired t-test. It is noticeable that PCC 11801 has a higher growth rate at 1% CO_2_ compared to 0.04% CO_2_ in SF conditions (Fig. 2D). The reduction in cell size may be attributed to the regulatory mechanisms of the cell that allows efficient uptake of nutrient by increasing surface area to volume ratio^32^. Furthermore, the reduction in cell size also allows efficient utilization of intracellular metabolite pools, lowers the thickness of the diffusion boundary layer and maximizes the availability of membrane transporters for facile nutrient uptake^32^.

The cell surface morphology was studied using cryo-SEM (Scanning Electron Microscopy). It was observed that PCC 11801 cells secrete a large amount exopolysaccharides (EPS) which covers the cell surface (Fig. 4C). The presence of copious amount EPS has also been demonstrated in the model strain *Synechocystis* sp. PCC 6803^33^. EPS have been depicted as the barrier to metal toxicity in cyanobacteria and protection against salt stress^33,34^. The presence of EPS in PCC 11801 suggests that the strain has evolved to acquire a protective mechanism against the salt and metal stress. This may be one of the reasons why PCC 11801 can tolerate and grow at sea salt concentrations.

### Genome Annotation and Comparative Genome Analysis

**Figure 5:**
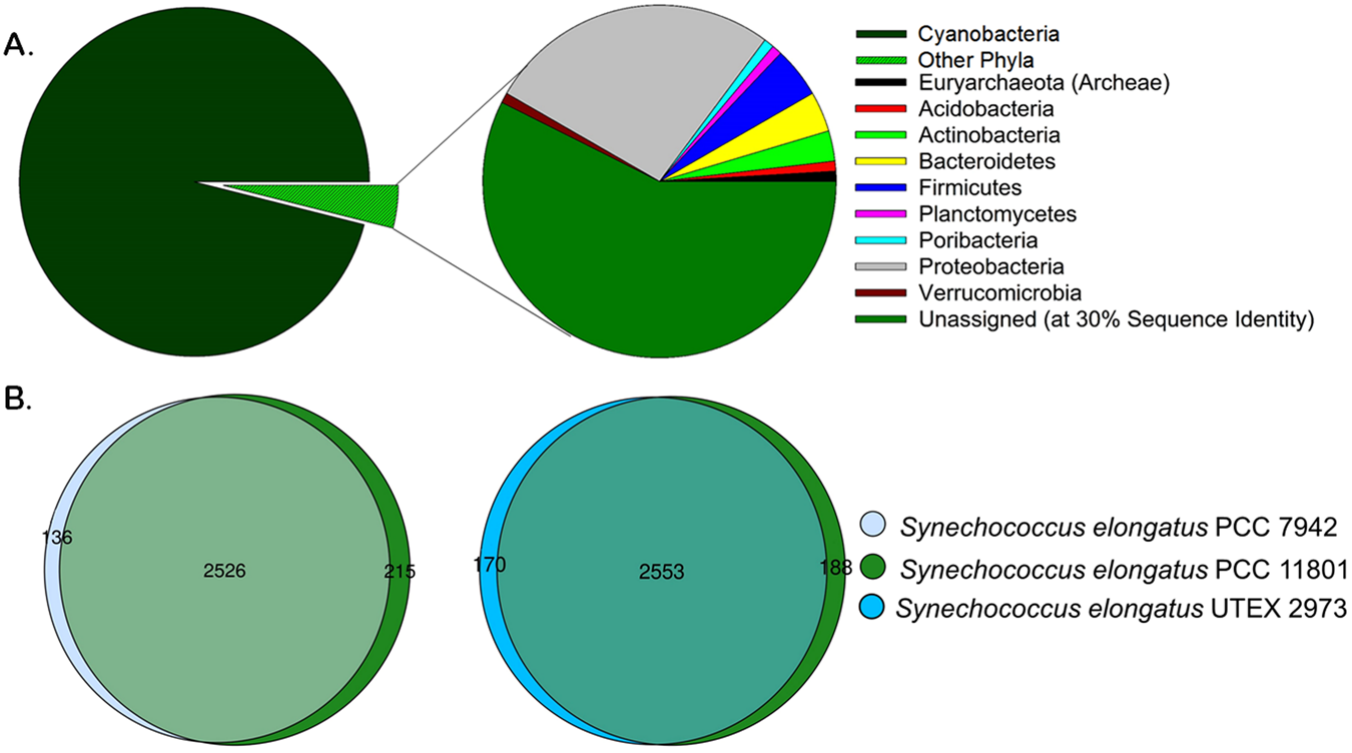
Comparative genome analysis of *Synechococcus elongatus* PCC 11801. (**A**) Gene distribution of PCC 11801 in different phyla and (**B**) Venn diagrams showing common genes between *Synechococcus elongatus* PCC 11801 and it’s closest neighbors PCC 7942 and UTEX 2973 at an expect value threshold of 10^−5^ and identity threshold of 30%.

The whole genome sequence of PCC 11801 reveals genome size of 2.7 Mbp. The general features of annotated genome of PCC 11801 obtained using IMG and RAST are shown in Table 1 (refer to Supplementary File S1 for complete annotation). The phylogenetic analysis (Fig. 1B) and genome comparison revealed that the closest neighbors of PCC 11801 are *S*. *elongatus* PCC 7942 and UTEX 2973. Unlike *S*. *elongatus* PCC 7942 and UTEX 2973 which are 99.8% identical at the genome level, the genome of PCC 11801 shows a query coverage of 90% and identity of 83% with its closest neighbor, *S*. *elongatus* PCC 7942 (Figs S3 and S4). The genome identity of other model cyanobacteria with their closest neighbor was found to be >90% (Table S3). Thus, PCC 11801 adds a significant genetic diversity to the group of completely sequenced cyanobacteria at the genome level. We also analyzed the gene distribution of PCC 11801. We found that 96% of the genes belonged to the cyanobacterial phylum and 2.3% were horizontally transferred from other phyla and rest remain unassigned (Fig. 5A and Supplementary File S1). Among the 96% genes that belonged to cyanobacterial phylum, a majority were assigned to order Synechococcales (2586) and others to Chroococcales (5), Chroococcidiopsidales (3), Nostocales (13), Oscillatoriales (21), Pleurocapsales (4) and Spirulinales (1) (Supplementary File S1).

**Table 1:** Genome annotation of *Synechococcus elongatus* PCC 11801 obtained using IMG^*^ and RAST^#^ with described criteria^†^.

	IMG	RAST
*Total gene count*	2793	2843
*Protein coding genes*	2741	2804
*GC Content*	54.9	54.9
*RNA coding genes*		
*rRNA*	2	—
*tRNA*	39	39
*Other RNA*	11	—
*Proteins associated with known function*	2121	2086
*Hypothetical*	620	718
*No homolog in S*. *elongatus PCC 7942*	215	344
*Homolog in S*. *elongatus UTEX 2973 but no homolog in S*. *elongatus PCC 7942*	38	42
^*^Integrated Microbial Genomes and Microbiomes, *JGI*^54^		
^#^ *Rapid Annotation using Subsystem Technology* ^56– 58^		
^†^ *E-value threshold of 10* ^*−5*^ *and identity threshold of 30%*

The whole genome alignment between PCC 11801 and its closest neighbor, PCC 7942 showed more than 0.4 million nucleotide polymorphisms (SNPs) (Fig. S5 and Supplementary File S2). Further, the gene-by-gene comparison between PCC 11801, PCC 7942 and UTEX 2973 shows 2515 proteins or ~90% of the genes to be common among them. We find 215 and 188 proteins unique to PCC 11801 as compared to PCC 7942 and UTEX 2973, respectively, at an expect value (E-value) threshold of 10^−5^ and identity threshold of 30% (Fig. 5B and Supplementary File S1). As the growth rate of this Indian isolate is comparable to UTEX 2973, we also investigated the proteins of PCC 11801 that had a homolog in UTEX 2973 but not in PCC 7942 (Table 1 and Supplementary File S1).

A major proportion of the genes in PCC 11801 that do not have a homolog in PCC 7942 code for proteins that allow adaptation to different kinds of stress conditions. The available functional annotation of these proteins is listed in Table 2. Previous reports have shown that the expression of these proteins in other organisms has resulted in stress-tolerant phenotypes. The deletion mutants, on the other hand, resulted into loss of function or a stress-sensitive phenotype. The presence of these genes in PCC 11801 thus implies its robust phenotype and that it can potentially serve a good model to study adaptive evolution. The genes coding for proteins involved in uptake and metabolism of sulfate esters were also found (Table 2).

**Table 2:** The unique genetic attributes of *Synechococcus elongatus* PCC 11801 compared to its closest neighbor, the model strain *Synechococcus elongatus* PCC 7942.

Protein	Function	Remarks based on literature reports
**Proteins related to stress tolerance**		
*Psb30 (Photosystem II Ycf12 subunit)*	Photosynthesis under high light	Deletion mutant became photosensitive and showed impaired growth at high light^60,61^
*Rubredoxin (Rd)*	Involved in Photosystem I and II activity	Deletion mutant shows functional loss of PSI activity in *Synechococcus* sp. PCC 7002^62^
*Tellurium resistance protein (Ter D)*	Impart resistance against tellurium	Deletion mutant of TerD is reported to be sensitive to tellurium compounds in *Streptomyces coelicolor*^63^
*ABC-type iron transport system protein (Fet AB)*	Iron export protein system	The overexpression of these proteins has been reported to abolish the peroxide sensitivity in *E*.*coli*^64^
*Glyoxylase like metal-dependent hydrolase*	Detoxification of methyl glyoxal	Upregulated under ethanol stress conditions in *Synechocystis* sp. PCC 6803^65^
*Alkylhydroperoxidase (Ahp D)*	Combat oxidative stress Mycobacterium sp.^66^	Expression of AhpD from *Anabaena* sp. PCC 7120 in *E*. *coli* showed tolerance and increased growth under different stress conditions like H_2_O_2_, CdCl_2_, UV etc^67^
*Bacterial capsule synthesis protein (PGA-cap)*	Production of poly gamma glutamate (PGA)	Helps in the survival of the organism under different stress conditions like high salt concentrations^68^.
*Sulfide quinone reductase*	Anoxygenic photosynthesis	Presence of this protein has been correlated in adaptation to sulfide toxicity in cyanobacteria^69^.
**Proteins related to sulfur metabolism**		
*Alkanesulfonate monooxygenase*	Uptake and metabolism of organosulfonates and arylsulfate esters	These proteins are reported to be active under sulfur limited condition in gram-negative bacteria^70,71^
*Alkanesulfonate transporter permease*		
*Arylsulfatases*		
*Formylglycine generating enzyme*		

Among the 215 proteins^†^ that are present in PCC 11801 but absent in PCC 7942, a few representative proteins with available functional annotation are listed here.

^†^E-value threshold of 10^−5^ and identity threshold of 30%.

Interestingly, we found that the genes coding for N-acetyltransferase family were more abundant in PCC 11801 than in PCC 7942. Some of these do not have any homolog in PCC 7942. N-Acetyltransferases are responsible for post-translational modification of proteins and thus are involved in gene regulation in eukaryotes and prokaryotes. They are reported to be involved in cell tolerance towards various environmental stress^35–37^. Additionally, a specific gene coding for O-acetyl-ADP ribose deacetylase that is a negative regulator of RNAase III and is involved in gene silencing and RNA maturation^38^ was also annotated in PCC 11801. Some genes coding for various proteins involved in type II toxin-antitoxin system and DNA repair like PIN domain nuclease, HEPN domain-containing protein, death-on-curing protein, HigAB, and HIRAN were also found in PCC 11801^39^. These proteins mediate gene regulation in response to various environmental stimuli and DNA repair post-replication. The presence of novel acetyltransferases, deacetylases, and other aforementioned proteins might play a crucial role in gene regulation and phenotype switching under extreme or stressful environmental conditions.

WD domain-containing protein that is highly studied and abundant in eukaryotes but rarely present in prokaryotes (6.5% of total)^40^ was also annotated in the genome of PCC 11801. WD proteins have conserved tryptophan-aspartate domain (WD) with a propeller structure consisting of 7 diverse repeating units. These proteins are involved in cell signaling, transcriptional regulation, ubiquitin-mediated protein degradation and chromatin modification in eukaryotes^41^. However, their function in prokaryotes remains unexplored. Recently, a study by *Hu*. *et al*. reported the comprehensive characterization of bacterial WD proteins^40^. Gene neighborhood analysis shows that the neighboring genes of WD proteins code for serine/threonine kinases and that it is not broadly conserved within cyanobacterial phylum but could be conserved within family^40^. The neighbor genes of WD protein were annotated as cobalamin (vitamin B_12_) synthesis protein in *Nostoc* and *Anabaena sp*^40^. The WD protein of PCC 11801 is homologous to *Nostoc* and *Anabaena* sp. and thus might be involved in cobalamin biosynthesis.

The PCC 11801 genome also contains a gene coding for enzyme, dibenzothiophene monooxygenase that detoxifies dibenzothiophene (DBT) into its oxygenated and desulfurized form. DBT forms a major source of sulfur in fossil fuels, and combustion of these fuels lead to air pollution. A previous study claimed to fight this problem by using dibenzothiophene monooxygenase containing bacterium *Achromobacter* sp. to bring about desulfurization of DBT^42^. Further investigations could be performed on PCC 11801 to check the activity of this enzyme. Thus, PCC 11801 could be a potential candidate for the development of the eco-friendly environment.

### Genetic manipulation of *Synechococcus elongatus* PCC 11801

**Figure 6:**
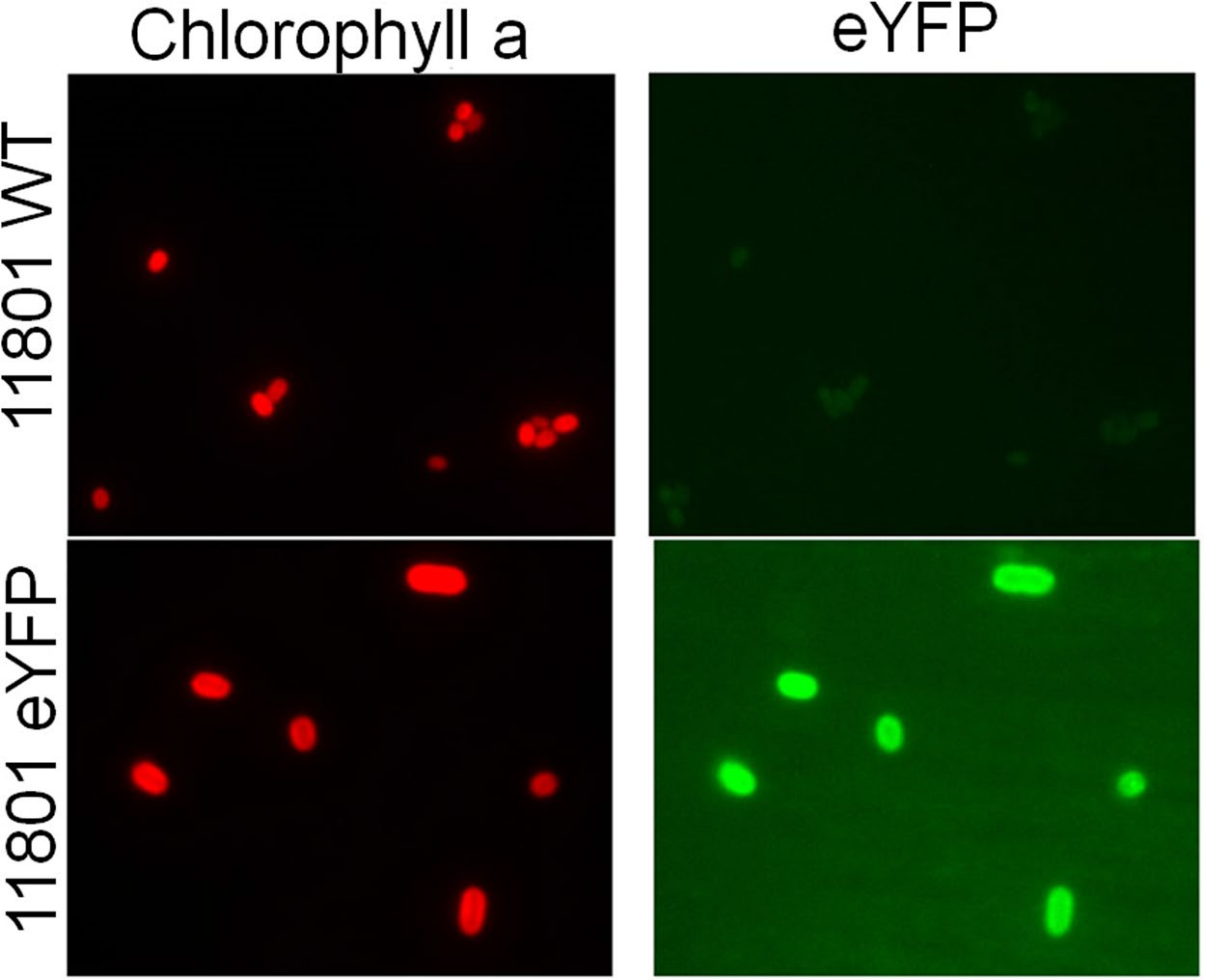
Genetic manipulation of *Synechococcus elongatus* PCC 11801. Microscopic images showing chlorophyll a fluorescence (left panels) and eYFP fluorescence (right panels) of WT and enhanced yellow fluorescence protein (eYFP) transformed cells of PCC 11801.

Genetic amenability is the key requirement for a strain to be used as a host for metabolic engineering applications. Similar to widely used model strains PCC 7942, PCC 7002 and PCC 6803, the strain PCC 11801 is naturally competent. The ability of PCC 11801 to facile targeted genetic manipulation was demonstrated by integration of transgene encoding enhanced yellow fluorescent protein (eYFP) controlled by cpcB promoter of PCC 7942 using a suicide vector (pSYN1). The recombinant eYFP expressing cells of PCC 11801 emitted strong fluorescence compared to the wild-type strain (Fig. 6). Another interesting phenomenon we observed was the portability of integrative vectors in PCC 11801 that were designed for targeted manipulations in PCC 7942. This offers an advantage as the synthetic or native parts and vectors from the model strain PCC 7942 can potentially be tested in PCC 11801 which will reduce the initial time to construct these. The faster growth of PCC 11801 compared to the model cyanobacteria coupled with the ease of genetic manipulation makes it a suitable host for metabolic engineering applications.

## Conclusion

We isolated and characterized a robust, fast growing, heat, light, and CO_2_ tolerant naturally transformable fresh water cyanobacterium, *Synechococcus elongatus* PCC 11801 from Powai Lake, Mumbai, India. The growth rate of PCC 11801 is comparable to the fastest growing cyanobacterium, *S*. *elongatus* UTEX 2973 and higher than the model strains *S*. *elongatus* PCC 7942, *Synechococcus* sp. PCC 7002 and *Synechocystis* sp. PCC 6803^16^. The strain PCC 11801 has a growth rate of 0.29 h^−1^ at 41 °C and 1000 µmole photons.m^−2^.s^−1^, which is the highest reported for any cyanobacterium under ambient CO_2_ conditions. The strain is also capable of tolerating sea salt concentration and does not require any additional supplements like vitamins for growth. These characteristics confer additional advantage as the strain can be cultivated in seawater which is abundantly available thereby avoiding competition with freshwater. Additionally, minimal medium requirements would reduce the production cost significantly.

Phylogenetic analysis using 16S rRNA and conserved house-keeping proteins along with genome analysis revealed that the closest neighbor strains are PCC 7942 and PCC 6301 (or UTEX 2973)^16^. Our study shows that the strain PCC 11801 shares ~83% genome identity with *S*. *elongatus* PCC 7942 and UTEX 2973 and possesses many unique genes that confer adaptation to extreme environmental stress conditions. PCC 11801 has many genes whose homologs are present in members of other class of cyanobacterial phylum but not in PCC 7942. The genome of PCC 11801 thus harbors unique genetic attributes from the phylogenetically diverse group of cyanobacteria that collectively might be responsible for its distinct phenotype of fast growth, tolerance to a variety of abiotic stresses along with genetic amenability. These unique features indicate that PCC 11801 could serve as a suitable host under field conditions.

Unlike its fast-growing neighbor *S*. *elongatus* UTEX 2973 that is not naturally transformable, PCC 11801 can take up DNA naturally. Moreover, the rapid growth of the strain significantly reduces the time to achieve chromosomal segregation. Interestingly, we found that the vector designed for homologous recombination in S. *elongatus* PCC 7942 works equally well with PCC 11801. Our results suggest that fast growth of PCC 11801 along with its stress tolerance and genetic amenability makes it a potential candidate for serving as a model laboratory strain as well as an attractive host for a wide range of biotechnological applications.

## Methods

### Isolation and axenic purification of *Synechococcus elongatus* PCC 11801

Water samples were collected from Powai lake on June 2^nd^, 2015. The cultures were grown in a shaker at 120 rpm, 38 °C, ambient CO_2_ and a light intensity of 400 µmole photons.m^−2^.s^−1^ in BG-11 medium till sufficient growth was observed. The cultures were serially diluted a few times. To remove the contamination of other prokaryotes, 20 mL culture were dark adapted in a shaker for 1 hour. Before the dark adaptation, 100 µL of a 10^−3^ dilution of original cultures were plated on LB Agar plates and incubated at 37 °C overnight. The number of colonies on LB plates was counted. After an hour of dark adaptation, kanamycin was added to cultures to reach a final concentration of 5 µg/mL. The cultures were kept in dark with shaking at 37 °C overnight. Next, the cells were centrifuged at 8000 g and the pellet was washed with BG-11 medium to remove the traces of kanamycin and again resuspended in 20 mL of medium. 100 µL of 10^−3^ dilutions of re-suspended cultures were plated on LB Agar plates and incubated at 37 °C overnight and colonies were counted. The entire procedure was repeated till no colonies were observed in undiluted samples. In order to remove eukaryotic contamination, the culture was grown till it attained a sufficient biomass and then treated with increasing concentration of cycloheximide (5–20 µg/mL). They were incubated in a shaker under normal growth conditions with the light-dark cycle of 14:10 hours for 6 passages. The culture was streaked on a BG-11 plate, and a single colony of the reported strain was isolated. No fungal contamination was found.

### Growth conditions

The strain was maintained in a shaker at a temperature of 38 °C, 120 rpm, ambient (0.04%) CO_2_ and light intensity of 400 µmole photons.m^−2^.s^−1^ in BG-11 medium (pH = 7.5) for the entire duration of isolation and axenic purification. CO_2_ tolerance was studied in CO_2_ incubator shaker (Adolf Kuhner AG, LT-X, Birsfelden, Switzerland) at CO_2_ concentrations of 0.04%, 0.5%, 1%, 5%, 10% and 15% in the chamber at 38 °C. Growth at varying light, temperature, CO_2_ and salt tolerance (at concentrations of 250, 350, 500, 600 and 700 mM NaCl) was measured in terms of OD_720_ in a multi-cultivator (Photon Systems Instruments, MC 1000-OD, Czech Republic) with an inlet gas flow rate of 800 SCCM to bubble all 8 tubes of multi-cultivators. The specific growth rate (µ) was measured in early exponential phase (approx. 0.1–0.5 OD_720_ nm in case of experiments in multi cultivators and 0.2–0.8 OD_720_ in SF) from the slope of semi-logarithmic plot between OD_720_ nm versus time. Doubling time was calculated as ln (2)/µ.

### Whole cell absorbance spectrum

The cell absorption spectrum was measured from 300–750 nm using UV-visible spectrophotometer (Shimadzu, UV-2600, Singapore) with one mL of culture.

### Phylogenetic analysis

The genomic DNA was isolated using a previously described protocol^43^. The DNA sequence corresponding to 16S rRNA was amplified from the genomic DNA with specific forward and reverse primers reported earlier^44^. The PCR products were enzymatically purified using Exo-SAP (Exonuclease I-Shrimp Alkaline Phosphatase) to remove extra dNTPs and primers by incubating at 37 °C for 60 minutes followed by 80 °C for 15 minutes. The nucleotide sequences of purified products were determined by capillary Genetic Analyzer 3500XL (Life Technologies). The obtained sequence was aligned with 16S rRNA sequences of 96 other organisms representing different clades^24^ using multiple sequence alignment tool, Clustal Omega^45^. The guide tree file obtained was used to construct a phylogenetic tree using iTOL^46^ (Interactive Tree Of Life).

A set of 29 phylogenetic marker proteins described previously^23,25^ was used to study the evolutionary relatedness of PCC 11801 with 130 other organisms. The concatenated sequences of these 29 proteins (DnaG, Frr, NusA, Pgk, PyrG, RplA, RplB, RplC, RplD, RplE, RplF, RplK, RplL, RplM, RplN, RplP, RplS, RplT, RpmA, RpoB, RpsB, RpsC, RpsE, RpsI, RpsJ, RpsK, RpsM, RpsS, and SmpB) were obtained from CyanoGEBA^24^ resource. The respective protein sequences from PCC 11801 were concatenated and aligned with those of 130 organisms, and the phylogenetic tree was constructed using MEGA7 and iTOL^46^.

### Biochemical estimations

The chlorophyll, carotenoids, carbohydrates and glycogen content were measured using cells harvested at 0.7 OD_720_ nm at different CO_2_ concentrations (0.04%, 0.5%, 1%, 5% and 10%), 38 °C, 400 µmole photons.m^−2^.s^−1^ and 120 rpm. Chlorophyll and carotenoid extraction was performed by a procedure described previously^47–49^. Briefly, two mL of culture was centrifuged at 8000 g for 7 minutes at 4 °C. One mL methanol (precooled at 4 °C) was added to the pellet. The suspension was vortexed, covered with aluminum foil and incubated at 4 °C for 20 minutes for cold extraction of pigments. The sample was again centrifuged, and the absorbance of the supernatant was measured at the wavelengths of 470, 665 and 720 nm against methanol as blank. The concentrations of chlorophyll and carotenoids were then calculated as:

1 $$Chla\,\,(\frac{\mu g}{ml})=12.9447\,({A}_{665}-{A}_{720})$$

2 $$Carotenoids\,(\frac{\mu g}{ml})=[1000({A}_{665}-{A}_{720})-2.86\,(\mathrm{Chla}\,(\mu g/{\rm{mL}})]/221$$

For total carbohydrate estimation, two mL of culture was centrifuged at 8000 g for 7 minutes at 4 °C. The pellet was reconstituted in 200 µL of autoclaved milli-Q water. Next, 200 µL of 5% phenol (w/v) in water was added followed by addition of 1 mL of ice-cold sulphuric acid. The solution was mixed well by inverting three times and incubated at room temperature for 10 minutes. Further, the samples were incubated at 35 °C for another 20 minutes. Finally, the absorbance was measured at 490 nm against a blank containing all the reagents except the biomass. The biomass samples were diluted depending on the content under varying conditions. The amount of carbohydrate was calculated using a standard plot of absorbance (A_490_) versus various glucose concentrations estimated using the same method.

The glycogen content was estimated using 10 mL of culture^50^. The cells were centrifuged at 8000 g for 10 minutes at 4 °C. One mL of precooled methanol was added to the pellet and vortexed. The suspension was incubated at 60 °C for 15 minutes, cooled at room temperature and again centrifuged at 8000 g for 10 minutes. The resulting pellet was washed with 100% ethanol. Next, 100 µL of 40% KOH was added to the pellet and incubated at 95 °C for an hour to remove free glucose. Then, 200 µL of 100% ethanol was added to the solution after cooling and kept at −20 °C overnight to precipitate glycogen. Next, the samples were centrifuged for 1 hour at 13000 g followed by addition of 40 µL of 2 N HCl and incubation at 95 °C for 30 minutes. The sample was cooled to room temperature, and 40 µL of 2 N NaOH, 20 µL of 1 M phosphate buffer (pH = 7.0) and 40 µL of autoclaved milli-Q water was added. The sample was vortexed thoroughly. Twenty µL of this sample was added to 1 mL of glucose oxidase enzyme-based glucose assay solution (GOD-PAP, Biolab Diagnostics, India), covered with aluminum foil and incubated at 37 °C for 15 minutes. Finally, the absorbance at 500 nm was measured. The amount of glycogen was calculated using a standard plot of OD_500_ versus varying glycogen concentrations estimated using the same method.

The amount of chlorophyll, carotenoids, total carbohydrates and glycogen content obtained in µg or mg was normalized with the biomass (mg) to obtain the respective content in µg/mg or mg/mg of dry cell weight.

### Transmission Electron Microscopy

Electron microscopy was performed on the cells harvested from two different growth conditions in SF, 0.04% and 1% CO_2_. The cells were centrifuged at 8000 g for 3 minutes, and the pellet was washed three times with milli-Q water. The cell pellet was fixed with 3% glutaraldehyde solution for 2 hours at 4 °C. Further, the cells were washed three times with 0.1 M sodium cacodylate buffer for 10 minutes. Cell fixation was done with osmium tetraoxide for 1 h at 4 °C followed by washes with 0.1 M sodium cacodylate buffer for 10 minutes. The cells were sequentially dehydrated with 50%, 70%, 90% and absolute alcohol for 15, 30, 15 and 60 minutes respectively. Ultra-thin sections of 60–80 nm were made and stained with uranyl acetate and lead citrate. Imaging was performed using JEOL 1400 Plus TEM,120 Kv (Tokyo, Japan).

### Scanning Electron Microscopy (Cryo-SEM)

One mL of exponentially growing cells were harvested by centrifugation at 8000 g and washed thrice with distilled water. The cells were reconstituted in 200 µL distilled water and analyzed using cryo FEG-SEM recorded on a JEOL JSM-7600F microscope (Akishima, Japan) under cryo mode.

### Whole genome sequencing and genome analysis

The extracted genomic DNA was treated with RNAase to remove RNA contamination. The whole genome was sequenced using the Ion Torrent Personal Genome Machine (PGM) system (Life Technologies). The quality check of the raw reads was performed using FastQC. The raw reads were assembled to contigs using SPAdes^51^, and the quality assessment of assembled reads was performed by QUAST^52^. Reference-guided assembly of the genome was done using ABACUS^53^ taking *Synechococcus elongatus* UTEX 2973 as a reference genome. The additional gaps in the genome were filled using PCR using specific primers, and the whole genome sequence was submitted in GenBank (accession number: CP030139). The assembled whole genome sequence of PCC 11801 was annotated using IMG^54,55^ (IMG-ID: 2770939469) and Rapid Annotation using Subsystem Technology (RAST)^56–58^.

### Genetic Manipulation

PCC 11801 was transformed using integrative vector pSYN1 containing neutral site 1 (NS1)^59^ carrying eYFP gene under cpcB promoter from *S*. *elongatus* PCC 7942. Five mL of 0.6 OD_730_ culture of PCC 11801 was centrifuged at 14,000 g for 3 minutes at room temperature. The pellet was washed and re-suspended in 100 µL BG-11. To this, 100 ng pSYN1 plasmid was added. Empty integrative vector was used as a negative control in parallel. The cell-DNA mixture(s) was incubated at 38 °C in the dark overnight. After the incubation was complete, the mixture was transferred to 20 mL BG-11 media containing 10 µg/mL spectinomycin. The culture was incubated at 38 °C for the growth of the transformants. Antibiotic concentration was increased gradually to allow complete chromosomal segregation. Verification of complete segregation was checked using confirmation primers, 5′ CGAAGATGGAAAAGCTCAAGCGGAAGGGACCCAAC3′ and 5′ GCGATTTGGGTAGCGCTGCCTTCCCCTTC 3′.

### Fluorescence Microscopy

Cells (~1 OD_720_) from wildtype and eYFP mutant were centrifuged at 8000 g for 3 minutes. The pellet was washed with milli-Q water twice and then re-suspended in 4% paraformaldehyde for 30 minutes at 4 °C. The fixed cells were mounted on the slide and observed under a fluorescence microscope. All fluorescence images were acquired with a Zeiss Axio Observer Z1 (100X objectives, NA = 1.40; Carl Zeiss MicroImaging Inc., Oberkochen, Germany) equipped with Axiocam camera controlled by Axiovision software [Axio Vision Release 4.8.3 SP1 (06–2012)]. Exposure time for imaging was 300 ms.

## Electronic supplementary material

Supplementary Information

Supplemental File S-1

Supplemental File S-2

Supplemental File S-3

Supplemental File S-4

## Data Availability

All data generated or analyzed during this study are included in this article (and its Supplementary Information files). The complete genome and 16S rRNA gene of *Synechococcus elongatus* PCC 11801 is available at GenBank under accession numbers, CP030139 and MH469534, respectively.
