# Supplementary material for: Genome Features and Biochemical Characteristics of a Robust, Fast Growing and Naturally Transformable Cyanobacterium Synechococcus elongatus PCC 11801 Isolated from India
Source: Sci Rep. 2018 Nov 9;8:16632. doi: 10.1038/s41598-018-34872-z (PMC6226537; doi:10.1038/s41598-018-34872-z)
Supplement: Supplementary file 1 — Supplementary Information [file 41598_2018_34872_MOESM1_ESM.pdf]

Supplementary Information

Genome Features and Biochemical Characteristics of a Robust, Fast Growing and Naturally Transformable Cyanobacterium *Synechococcus elongatus* PCC 11801 Isolated from India

Damini Jaiswal[1], Annesha Sengupta[1], Sujata Sohoni[1], Shinjinee Sengupta[1,2], Ambarish G. Phadnavis[1], Himadri B. Pakrasi[3,4,] and Pramod P. Wangikar[1,2,5*]

[1]*Department of Chemical Engineering, Indian Institute of Technology Bombay, Powai, Mumbai 400076, India*

[2]*DBT-PAN IIT Centre for Bioenergy, Indian Institute of Technology Bombay, Powai, Mumbai 400076, India*

[3]*Department of Biology, Washington University, St. Louis, MO 63130*

[4]*Department of Energy, Environmental and Chemical Engineering, Washington University, St. Louis, Missouri, USA*

[5]*Wadhwani Research Centre for Bioengineering, Indian Institute of Technology Bombay, Powai, Mumbai 400076, India*

[*]*Corresponding author email: wangikar@iitb.ac.in*

Table of Contents

Figure S1	S-3
Figure S2	S-4
Figure S3	S-5
Figure S4	S-5
Figure S5	S-6
Table S1	S-6
Table S2	S-7
Table S3	S-9
Supplemental Discussions	S-9
Supplemental Files	S-10
References	S-11

Figure S1: The phylogenetic tree constructed based on 16S rRNA sequences showing the lineage of *Synechococcus elongatus* PCC 11801. Nodes supported by bootstrap values of > 0.7 are indicated.

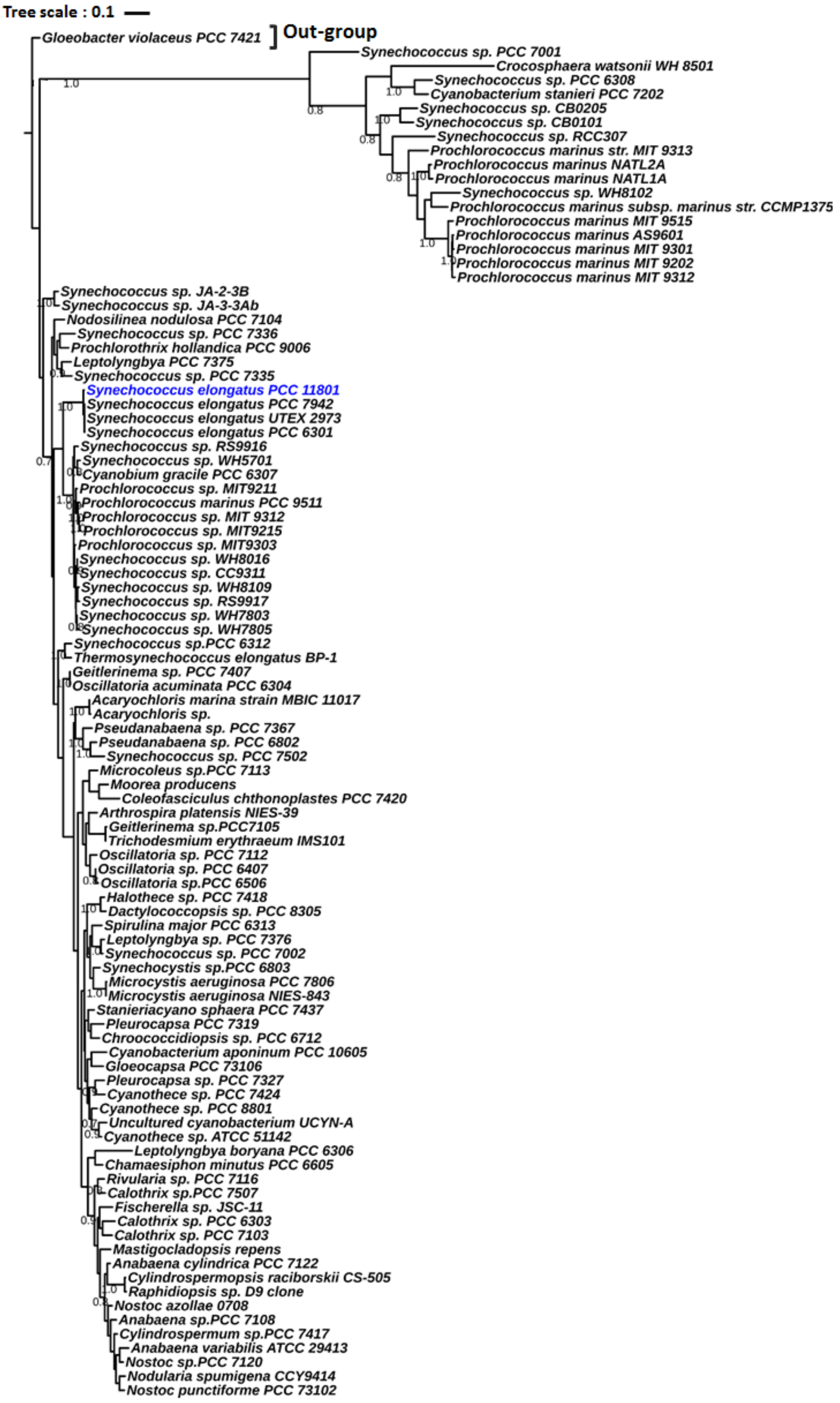

Figure S2: The phylogenetic tree constructed using concatenated sequences of 29 house-keeping proteins showing the lineage of *Synechococcus elongatus* PCC 11801. Nodes supported by bootstrap values of > 0.7 are indicated.

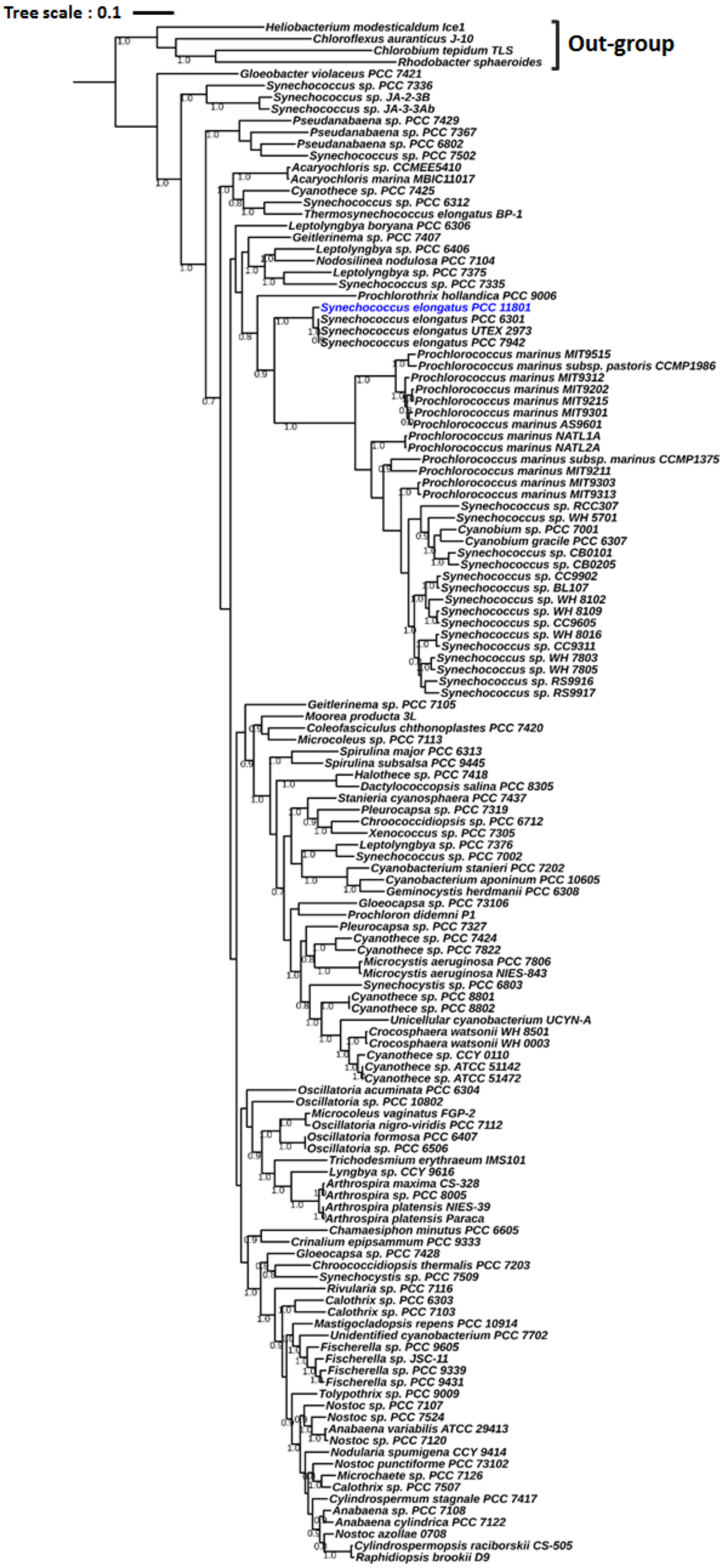

Figure S3: The dot plot between *Synechococcus elongatus* PCC 7942 and UTEX 2973

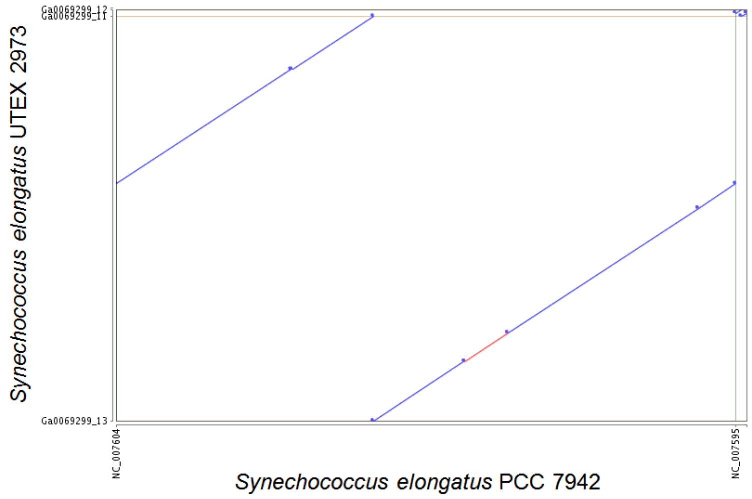

Figure S4: The dot plot between *Synechococcus elongatus* PCC 7942 and *Synechococcus elongatus* PCC 11801

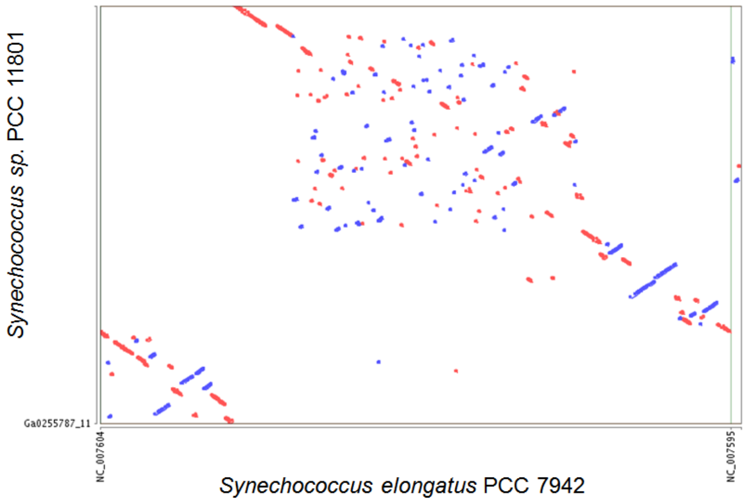

Figure S5: The whole genome alignment of *Synechococcus elongatus* PCC 11801 with *Synechococcus elongatus* PCC 7942 as reference

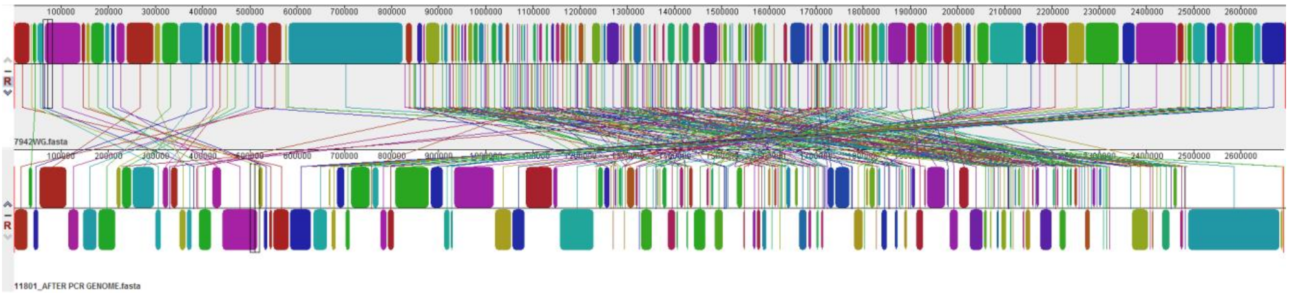

Table S1: Doubling times (in hours) of *Synechococcus elongatus* PCC 11801 under different conditions. Highlighted value refers to the least doubling time obtained for PCC 11801.

			Light Intensity (µmole photons.$m^{-2}.s^{-1}$)				
Temperature		**CO_2 (v/v)**	200	400	600	800	1000
	43	0.04%	-	8.3 ± 2.7	6.4 ± 0.1	5.6 ± 0.1	5.5 ± 0.2
	41	0.04%	-	4.7 ± 0.4	3.4 ± 0.2	2.7 ± 0.1	2.3 ± 0.1
		1%	-	6.1 ± 0.5	4.0 ± 0.1	4.9 ± 0.2	4.9 ± 0.1
	38	0.04%	14.8 ± 0.1	5.8 ± 0.3	4.1 ± 0.4	3.4 ± 0.4	2.7 ± 0.2
		1%	-	6.9 ± 0.5	4.8 ± 0.3	4.5 ± 0.3	3.3 ± 0.4
		3%	-	7.5 ± 1.4	7.6 ± 0.0	6.9 ± 0.0	6.5 ± 0.2
	33	0.04%	-	7.4 ± 0.3	5.27 ± 0.7	4.1 ± 0.1	5.7 ± 0.7

Table S2: Individual cell length measurements for *Synechococcus elongatus* PCC 11801 under 0.04 % and 1 % CO_2 conditions

	Length (nm)	
S.No.	0.04% CO2	1% CO2
1	2601.2	1166.3
2	2501.6	1972.6
3	2481.6	745.1
4	2388.2	1644.0
5	2746.9	985.2
6	1674.7	1766.8
7	1782.9	1216.3
8	1623.3	1215.5
9	2555.9	980.1
10	2430.0	1749.5
11	2407.6	830.5
12	2369.2	1891.7
13	2326.9	979.1
14	2343.7	754.1
15	2302.2	2286.9
16	2409.3	1658.9
17	2772.2	1650.2
18	1851.8	1041.1
19	2838.2	786.6
20	2777.6	1463.3
21	2605.8	1439.3
22	1899.9	1505.2
23	1894.3	1508.3
24	2784.1	1255.1
25	2035.6	1190.0
26	2795.1	1255.2
27	2160.4	1268.6
28	2398.9	1766.9
29	2757.5	1068.5
30	3457.1	1283.5
31	2253.3	833.3
32	2486.8	996.0
33	2309.1	770.1
34	2270.5	1615.8
35	2637.8	1108.7
36	2446.3	1234.4
37	2498.5	1650.0
38	3625.0	1809.2
39	2943.1	1504.8
40	3217.0	1088.8
41	2877.9	944.7
42	2403.5	1306.8
43	2191.6	790.1
44	2360.8	878.2
45	2405.2	1185.2

46	2142.0	1892.2
47	1881.6	1773.4
48	1892.4	1186.3
49	3271.6	658.4
50	2788.3	1506.5
51	2243.6	1097.7
52	2379.2	811.0
53	2386.2	817.3
54	2122.1	1747.0
55	1974.9	1545.9
56	3079.8	1571.0
57	3499.7	2081.3
58	2917.6	1385.8
59	3206.5	852.2
60	3281.8	1121.7
61	2852.8	1099.5
62	3280.6	1411.2
63	2925.0	1402.6
64	2817.1	1269.2
65	2655.9	1159.8
66	2302.0	1728.0
67	1887.1	2221.1
68	2339.7	748.2
69	1820.9	1073.8
70	-	847.8
71	-	947.0
72	-	1671.6
73	-	1649.5
74	-	2843.8
75	-	1144.3
76	-	2038.6
77	-	2597.3
Average length (nm)	2505.1	1349.9
Average length (µm)	2.5	1.3

Table S3: The genome identity of the model cyanobacterial strains with their closest neighbors

Query	Nearest cyanobacteria neighbour	Query Coverage	E-value	% Identity
***Synechococcus elongatus* PCC 11801**	*S. elongatus PCC 7942*	90%	0	83%
	S. elongatus UTEX 2973	90%	0	83%
***Synechococcus elongatus* PCC 7942**	*S. elongatus PCC 6301*	100%	0	99%
	S. elongatus UTEX 2973	99%	0	99%
***Synechococcus* sp. PCC 7002**	*Synechococcus sp. PCC 8807*	95%	0	98%
	Synechococcus sp. PCC 7117	95%	0	97%
***Synechocystis* sp. PCC 6803**	*Synechocystis sp. PCC 6714*	76%	0	90%
***Synechococcus elongatus* PCC 6301**	*S. elongatus UTEX 2973*	99%	0	99%
	S. elongatus PCC 7942	99%	0	99%

Supplemental Discussion

Proteins conferring stress adaption in PCC 11801

We found several proteins in PCC 11801 that are known to confer adaptive advantage under different stress conditions and do not have a homolog in PCC 7942. These proteins include photosystem II complex subunit Ycf12 (Psb30), rubredoxin (rd), tellurium resistance protein (TerD), ABC-type iron transport system (FetAB), glyoxylase like metal-dependent hydrolase, alkylhydroperoxidase (AhpD), bacterial capsule synthesis protein (PGA-cap) and sulfide-quinone reductase.

Psb30 or Ycf12 protein is present both in UTEX 2973 and PCC 11801 and does not have a homolog in PCC 7942. Psb30 plays an important role during photosynthesis under high light conditions. The deletion mutant of Psb30 became photosensitive and showed impaired growth at high light[1,2]. Rubredoxin (Rd) is a soluble iron-containing protein involved in various biological processes like carbon fixation, fatty acid metabolism and detoxification of reactive oxygen species (ROS).[3–5] Rubredoxin has been shown to restore PSII activity in 2pac mutant of *Chlamydomonas* sp.[6]. A deletion mutant of rd has been reported to result in a functional loss of PSI activity in *Synechococcus* sp. PCC 7002[7]. Thus, rd is important for both PSI and PSII activity. The class of rubredoxins present in organisms performing oxygenic photosynthesis is distinct from rubredoxins present in another group of organisms. However, surprisingly, the rd protein in PCC

11801 showed the best hit with that of *Acidobacteria bacterium*. This implies that rd protein in PCC 11801 might have some additional functions similar to bacterial rubredoxins. TerD is responsible for imparting resistance against tellurium. The deletion mutant of TerD in *Streptomyces coelicolor* is reported to render it sensitive to tellurium compounds[8]. The homolog of this protein was found in PCC 7002.

FetAB is an iron export protein system that consists of FetA (ATP binding cassette) and FetB (iron permease). The iron transporter present in PCC 7942 (FutB) was not conserved with that of FetB found in PCC 11801. The best hit using NCBI BLAST was *Fischerella* sp. PCC 9339 and has a homolog in *Synechococcus* sp. PCC 7002 with a sequence identity of 43% but very limited information is available on these two proteins in cyanobacteria. The overexpression of these proteins has been reported to abolish the peroxide sensitivity in *E.coli* [9]. Glyoxylase like protein is responsible for detoxification of methyl glyoxal and helps in survival under adverse conditions. This protein was found to be upregulated under ethanol stress conditions in *Synechocystis* sp. PCC 6803[10]. AhpD family alkyl hydroperoxidase is found specifically in *Mycobacterium* sp. to combat oxidative stress[11]. The expression of AhpD from *Anabaena* sp. PCC 7120 in *E. coli* showed tolerance and increased growth under different stress conditions like H_2O_2, $CdCl_2$, UV etc[12]. PGA-cap produces poly gamma glutamate (PGA) which helps in the survival of the organism under different stress conditions like high salt concentrations[13]. Sulfide-quinone reductase is involved in anoxygenic photosynthesis where inorganic sulfides are electron donor. The presence of this protein has been correlated in adaptation to sulfide toxicity in cyanobacteria[14].

Supplemental Files

Supplemental File S-1: Complete Annotation, unique and common proteins between PCC 11801 and PCC 7942 and gene distribution of *Synechococcus elongatus* PCC 11801 obtained from IMG

Supplemental File S-2: Single Nucleotide Polymorphisms (SNPs) in *Synechococcus elongatus* PCC 11801 using *Synechococcus elongatus* PCC 7942 as a reference

Supplemental File S-3: All gene sequences of *Synechococcus elongatus* PCC 11801 in fasta format

Supplemental File S-4: All protein sequences of *Synechococcus elongatus* PCC 11801 in fasta format

References

1. Inoue-Kashino, N., Kashino, Y. & Takahashi, Y. Psb30 is a photosystem II reaction center subunit and is required for optimal growth in high light in *Chlamydomonas reinhardtii*. *J. Photochem. Photobiol. B Biol.* **104,** 220–228 (2011).

2. Inoue-Kashino, N. *et al.* Evidence for a stable association of Psb30 (Ycf12) with photosystem II core complex in the cyanobacterium *Synechocystis* sp. PCC 6803. *Photosynth. Res.* **98,** 323–335 (2008).

3. Kurtz, D. M. Microbial detoxification of superoxide: The non-heme iron reductive paradigm for combating oxidative stress. *Acc. Chem. Res.* **37,** 902–908 (2004).

4. Manyani, H., Rey, L., Palacios, J. M., Imperial, J. & Ruiz-Argüeso, T. Gene products of the hupGHIJ operon are involved in maturation of the iron-sulfur subunit of the [NiFe] hydrogenase from *Rhizobium leguminosarum* bv. viciae. *J. Bacteriol.* **187,** 7018–7026 (2005).

5. Fritsch, J., Lenz, O. & Friedrich, B. The maturation factors HoxR and HoxT contribute to oxygen tolerance of membrane-bound [NiFe] hydrogenase in *Ralstonia eutropha* H16. *J. Bacteriol.* **193,** 2487–2497 (2011).

6. Calderon, R. H. *et al.* A conserved rubredoxin is necessary for photosystem II accumulation in diverse oxygenic photoautotrophs. *J. Biol. Chem.* **288,** 26688–26696 (2013).

7. Shen, G. *et al.* Assembly of photosystem I. I. Inactivation of the rubA gene encoding a membrane-associated rubredoxin in the cyanobacterium *Synechococcus* sp. PCC 7002 causes a loss of photosystem I activity. *J. Biol. Chem.* **277,** 20343–54 (2002).

8. Sanssouci, É., Lerat, S., Grondin, G., Shareck, F. & Beaulieu, C. Tdd8: A TerD domain-encoding gene involved in *Streptomyces coelicolor* differentiation. *Antonie van Leeuwenhoek, Int. J. Gen. Mol. Microbiol.* **100,** 385–398 (2011).

9. Nicolaou, S. A., Fast, A. G., Nakamaru-Ogiso, E. & Papoutsakis, E. T. Overexpression of fetA (ybbL) and fetB (ybbM), encoding an iron exporter, enhances resistance to oxidative stress in *Escherichia coli*. *Appl. Environ. Microbiol.* **79,** 7210–7219 (2013).

10. Wang, J. *et al.* RNA-seq based identification and mutant validation of gene targets related to ethanol resistance in cyanobacterial *Synechocystis* sp. PCC 6803. *Biotechnol. Biofuels* **5,** 89 (2012).

11. Hillas, P. J., del Alba, F. S., Oyarzabal, J., Wilks, A. & Ortiz de Montellano, P. R. The AhpC and AhpD antioxidant defense system of *Mycobacterium tuberculosis* . *J. Biol. Chem.* **275,** 18801–18809 (2000).

12. Shrivastava, A. K., Singh, S., Singh, P. K., Pandey, S. & Rai, L. C. A novel alkyl hydroperoxidase (AhpD) of *Anabaena* PCC 7120 confers abiotic stress tolerance in *Escherichia coli* . *Funct. Integr. Genomics* **15,** 77–92 (2015).

13. Luo, Z. *et al.* Microbial synthesis of poly-γ-glutamic acid: current progress, challenges, and future perspectives. *Biotechnol. Biofuels* **9,** 134 (2016).

14. Cohen, Y., Jørgensen, B. B., Revsbech, N. P. & Poplawski, R. Adaptation to hydrogen sulfide of oxygenic and anoxygenic photosynthesis among cyanobacteria. *Appl. Environ. Microbiol.* **51,** 398–407 (1986).
